# Successful endovascular repair of complicated pseudoaneurysm using Perclose ProGlide: A novel concept

**DOI:** 10.1002/ccr3.6655

**Published:** 2022-11-27

**Authors:** Takahide Kodama, Tetsuo Yamaguchi, Hideomi Fujiwara, Masanari Kuwabara

**Affiliations:** ^1^ Department of Cardiology Toranomon Hospital Tokyo Japan

**Keywords:** closure device, complication, endovascular repair, Perclose ProGlide, pseudoaneurysm

## Abstract

Iatrogenic pseudoaneurysm is common vascular complications of angiographic procedures. Patients with uncomplicated pseudoaneurysms can be managed with ultrasound‐guided techniques. However, for complicated pseudoaneurysms, surgical repair of the artery is mandatory. We report a case of successful repair of complicated pseudoaneurysm using an access‐site closure device, Perclose ProGlide™ without a surgical approach.

## Graphical abstract

Management of a pseudoaneurysm with a Perclose ProGlideTM could be an option for the treatment when the pseudoaneurysm has a wide neck or complicated features, which is less invasive and costless.

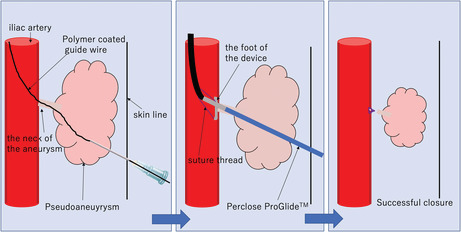

## 1 INTRODUCTION

Iatrogenic pseudoaneurysm is a false aneurysm that occurs after arterial wall injury related to an incomplete hemostatic plug at the puncture site. A pseudoaneurysm can develop at any arterial site used for arterial puncture. The most common site for pseudoaneurysm development is the femoral artery, which is used as access for percutaneous‐based diagnostic and interventional procedures. Uncomplicated pseudoaneurysms can usually be managed with ultrasound‐guided compression or percutaneous thrombin injection. However, for complicated pseudoaneurysms and those failing nonsurgical management, surgical repair of the artery is needed.

We herein report a case of successful repair of complicated pseudoaneurysm using an access‐site closure device, Perclose ProGlide™ (Abbott Vascular Inc.) without a surgical approach.

## 2 CASE PRESENTATION

**FIGURE 1:**
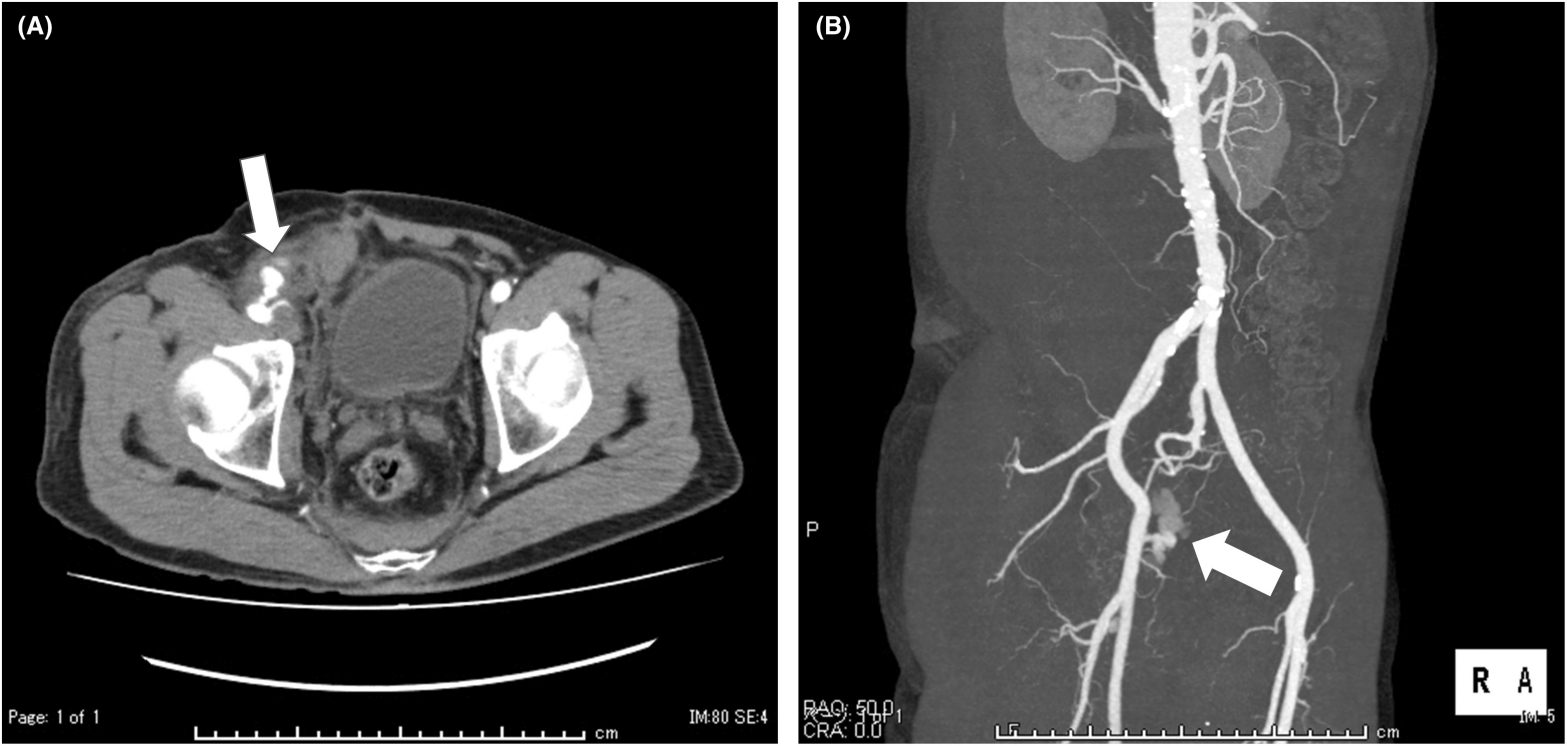
Contrast computed tomography showed that right‐sided retroperitoneal hematoma extending from the right femoral access site, suggesting pseudoaneurysm with extravasation (white arrow; (A) axial view and (B) 3D composition image)

A 72‐year‐old man consulted our division because of a femoral artery pseudoaneurysm. The patient had undergone endovascular treatment for left subclavian artery stenosis, for which the access site was the right femoral artery, 4 days before the consultation. Three days after the procedure, the patient complained of right lower quadrant pan, and a blood test showed a decreased hemoglobin (Hb) level from 12.9 to 9.0 g/dl. Computed tomography (CT) was then performed, revealing right‐sided retroperitoneal hematoma extending from the right femoral access site, suggesting a pseudoaneurysm with extravasation (Figure 1) (Video S1).

**FIGURE 2:**
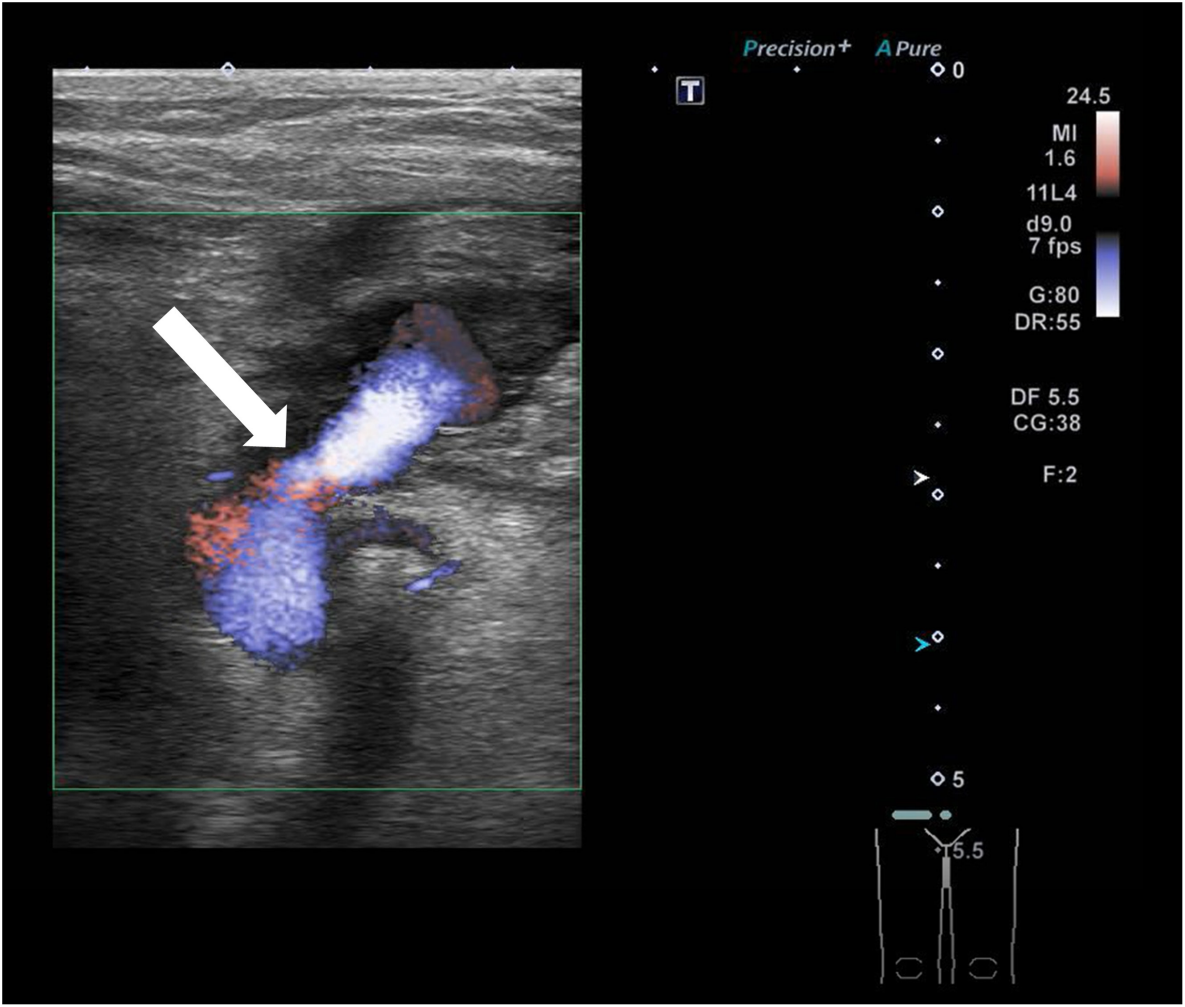
Duplex echo showed a pseudoaneurysm 40 mm in diameter, with an aneurysmal neck continuing from the femoral artery at 6.1 mm in diameter. White arrow shows blood flow from femoral artery to pseudoaneurysm

Although duplex echo‐guided compression was performed, follow‐up CT, the next day showed worsening retroperitoneal hematoma and progression of anemia (Hb 7.2 g/dl). On that day, systolic blood pressure temporarily dropped to 90 mmHg, but after a transfusion of red blood cells, blood pressure and heart rate stabilized at 122/78 mm Hg and 68 beats/min, respectively. Therefore, the previous team consulted our division. Duplex echo (Video S2) showed a pseudoaneurysm 40 mm in diameter, with an aneurysmal neck continuing from the femoral artery at 6.1 mm in diameter (Figure 2). We diagnosed this case as a complicated pseudoaneurysm with rupture.

**FIGURE 3:**
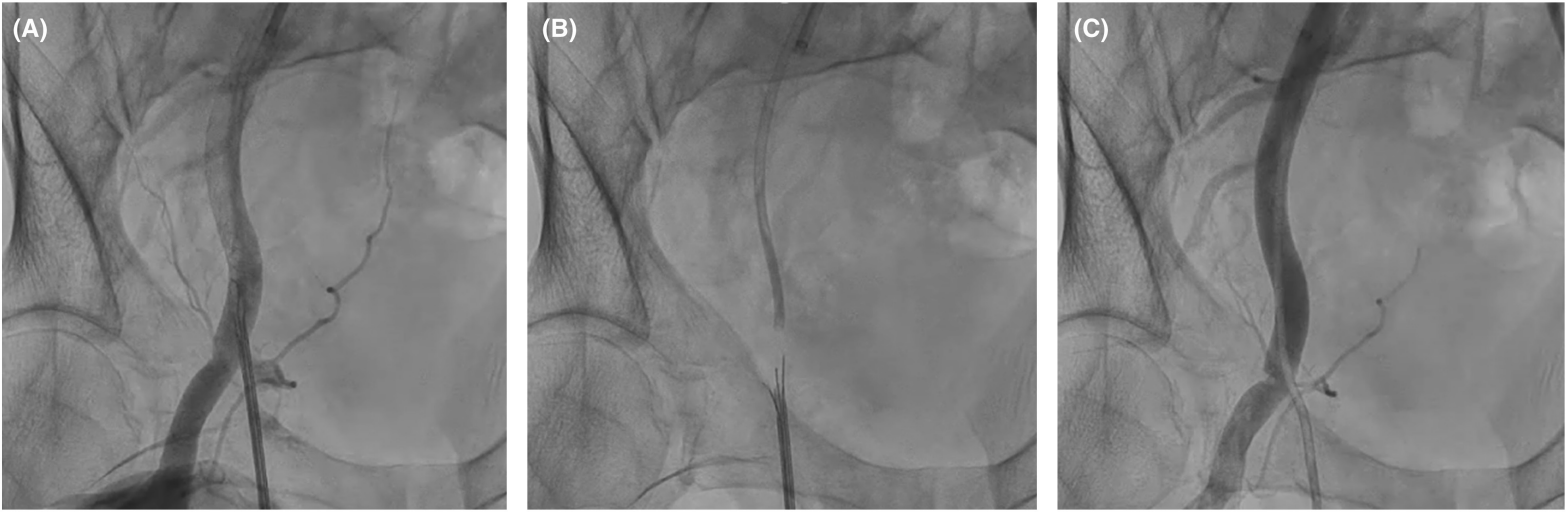
(A) Femoral angiography showed the pseudoaneurysm near the previously punctured skin mark. (B) The foot of Perclose ProGlide™ was deployed confirming that the position of the foot was in the true lumen of the iliac artery (inside of the vessel, not in the aneurysm) under fluoroscopy guidance. (C) Angiography showed the successful aneurysmal closure

**FIGURE 4:**
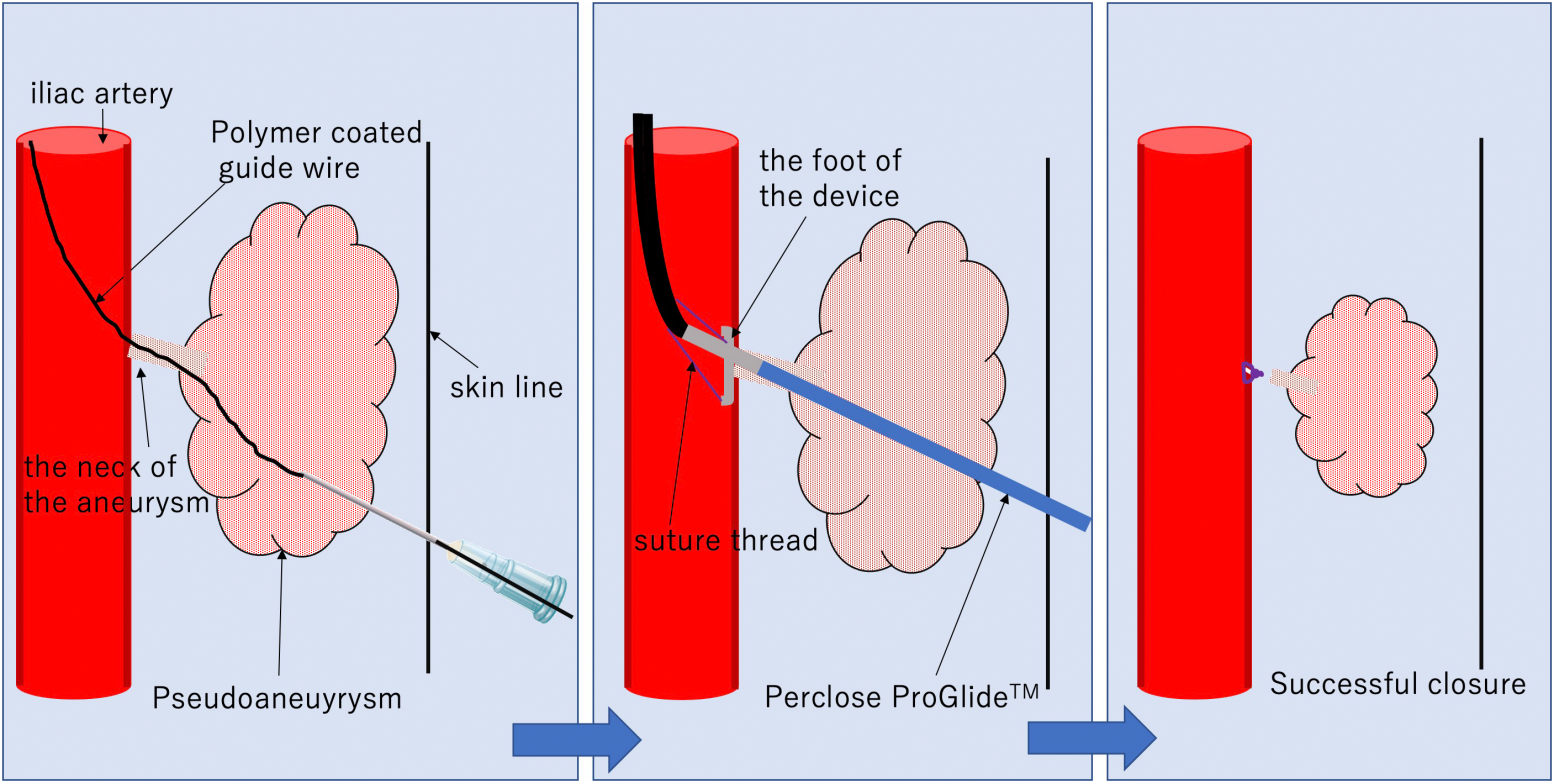
A schema of the mechanism of this procedure. Punctured the pseudoaneurysm just below the skin and then directly inserted the polymer‐coated guide wire into the true lumen of the iliac artery (left). Perclose ProGglide™ was tracked over the wire into the iliac artery, and the foot was deployed confirming that the position of the foot was in the true lumen of the iliac artery. When the device is pulled back against the vessel wall to deploy the sutures, bleeding stops suggesting successful closure is promising (mid). Successful closure and reduced size of the pseudoaneurysm (right)

Because there was no sign of infection and the patient condition was stable, we decided to perform endovascular repair using Perclose ProGlide™ on the cardiovascular surgery standby. To confirm the location of the pseudoaneurysm, we performed angiography using a contralateral femoral approach and punctured the pseudoaneurysm just below the skin near the previously punctured skin mark under duplex echo guidance. Subsequently, a 0.035‐inch polymer‐coated guidewire (Radifocus™, TERUMO, Tokyo) was directly inserted into the pseudoaneurysm. And then, we navigated the guidewire inside the pseudoaneurysm to cross the aneurysmal neck and get into the true lumen of the right iliac artery (Figure 4 left, Video S3). After getting the 0.035‐inch guidewire into the iliac artery, a 7‐Fr sheath was inserted to facilitate closure device insertion. After retrieval of the 7‐Fr sheath, Perclose ProGlide™ was tracked over the wire into the right iliac artery, and the foot was deployed confirming that the position of the foot was in the true lumen of the iliac artery (inside of the vessel, not in the aneurysm) under fluoroscopy guidance (Figures 3a,b and 4 center, Video S4). The closure was then performed, and successful aneurysmal closure was confirmed by angiography (Figures 3c and 4 right, Videos S5 and S6). The contralateral access site was also closed using the Perclose ProGlide™.

After the endovascular procedure, no recurrence of pseudoaneurysm was observed, and the patient was discharged on postoperative day 4.

## 3 DISCUSSION

Iatrogenic pseudoaneurysm is one of the most common vascular complications of cardiac and peripheral angiographic procedures. The incidence of pseudoaneurysm after interventional procedure ranges from 2% to 6%.^1^ Most patients with uncomplicated pseudoaneurysms can be managed with ultrasound‐guided techniques. Ultrasound‐guided thrombin injection (UGTI) is the first‐line technique, rather than ultrasound‐guided compression, because of the high success rate (97.5%) and acceptably low rate of thrombotic complications (0.5%).^2^ However, it should be noted that this is an off‐label use of thrombin.

For complicated pseudoaneurysm, defined as cases with hemodynamic instability, extensive skin, and subcutaneous damage, or soft tissue infection, the common treatment strategy is open surgical repair.^3^ The present patient showed progressive anemia and aneurysmal wall rupture, suggesting a complicated pseudoaneurysm. Due to the size and ruptured nature of the wall, coil embolization or UGTI was not suitable for this case. However, because there were no signs of infection, the management goal was simply to close the related arterial wall. Based on the duplex echo findings, we considered ultrasound‐guided puncture of the pseudoaneurysm and passage of a guidewire through the wide aneurysmal neck (6.1 mm) to be feasible. Perclose ProGlide™ was thus applied, and we adopted a low‐invasiveness strategy involving percutaneous suture with a Perclose ProGlide™ to successfully repair this case of a complicated large pseudoaneurysm. No recurrence has yet been noted.

In the literature, only one report has described two cases of successful pseudoaneurysm repair with Perclose ProGlide™.^4^ Although the previous report described the difficulties with this method, several tips would help overcome these problems. First, it is important to evaluate pseudoaneurysms and the neck using duplex echo to determine whether or not it is feasible to pass a guidewire through the aneurysmal neck. In addition, performing punctures near the previously punctured skin mark is essential. Second, angiography of the related lesion should be performed, and the aneurysmal neck should be visualized to facilitate guidewire passage. Third, to deploy the foot of the device at the appropriate position, confirmation by fluoroscopy guidance is vital. Usually bleeding from the indicator of the device indicates an intraluminal position of the foot of the device. However, as in the present case, bleeding from the indicator can be observed even when the foot of the device is inside the pseudoaneurysm, making it impossible to distinguish between the intra‐iliac artery and pseudoaneurysm. Therefore, the appropriate location of the foot must be confirmed under fluoroscopy.

We summarized 6 points of the indication for the management of a pseudoaneurysm with a Perclose ProGlide™; (1) hemodynamic stabilization is possible, (2) the pseudoaneurysm has a wide neck that a guidewire can pass, (3) there is no infectious sign, (4) difficult case to use thrombin injection, (5) it needs under cardiovascular surgery stand‐by, and (6) cardiovascular team discussion organized by both highly experienced cardiovascular physicians and surgeons should be held before the procedure.

## 4 CONCLUSION

Management of a pseudoaneurysm with a Perclose ProGlide™ could be an option for the treatment when the pseudoaneurysm has a wide neck or complicated features, which is less invasive and costless. To conduct this procedure, cardiovascular surgery standby is needed in case of the failure of the procedure, and it is also preferred that the patient condition is stable.

## AUTHOR CONTRIBUTIONS

The author Tetsuo Yamaguchi is an assistant operator of the procedure and preparation of figures. The author Hideomi Fujiwara is an assistant operator of the procedure and preparation of figures. The author Masanari Kuwabara involved in brief summary of the contribution.

## CONFLICT OF INTEREST

The authors declare no potential conflict of interest.

## CONSENT

Written informed consent was obtained from the patient to publish this report in accordance with the journal's patient consent policy.

## Supporting information


Video S1

Video S2

Video S3

Video S4

Video S5

Video S6

## Data Availability

The data that supports the findings of this study are available in the supplementary material of this article

## References

[1] Katzenschlager R , Ugurluoglu A , Ahmadi A , et al. Incidence of pseudoaneurysm after diagnostic and therapeutic angiography. Radiology. 1995;195:463‐466. 7724767 10.1148/radiology.195.2.7724767

[2] Webber GW , Jang J , Gustavson S , Olin JW . Contemporary management of postcatheterization pseudoaneurysms. Circulation. 2007;115:2666‐2674. 17515479 10.1161/CIRCULATIONAHA.106.681973

[3] Coley BD , Roberts AC , Fellmeth BD , Valji K , Bookstein JJ , Hye RJ . Postangiographic femoral artery pseudoaneurysms: further experience with US‐guided compression repair. Radiology. 1995;194:307‐311. 7824703 10.1148/radiology.194.2.7824703

[4] Liu W , Liu C , Lu SY . Percutaneous suture technique with ProGlide to manage vascular access pseudoaneurysm after percutaneous coronary intervention procedure: a case report. Chin J Traumatol. 2020;23:34‐37. 31956042 10.1016/j.cjtee.2019.11.002 PMC7049607

